# A competition model for prediction of admission scores of colleges and universities in Chinese college entrance examination

**DOI:** 10.1371/journal.pone.0274221

**Published:** 2022-10-28

**Authors:** Xiao Chen, Yi Peng, Yachun Gao, Shimin Cai

**Affiliations:** 1 School of Computer Science and Engineering, University of Electronic Science and Technology of China, Chengdu, Sichuan, China; 2 School of International Studies, Zhejiang University, Hangzhou, Zhejiang, China; 3 School of Physics, University of Electronic Science and Technology of China, Chengdu, Sichuan, China; Hanyang University, KOREA, REPUBLIC OF

## Abstract

Predicting the admission scores of colleges and universities is significant for high school graduates in the College Entrance Examination in China (which is also called “Gaokao” for short). The practice of parallel application for the students after Gaokao not only puts forward a question about how students could make the best of their scores and make the best choice, but also results in the strong competition among different colleges and universities, with the institutions all striving to admit high-performing students in this examination. However, existing prevailing prediction algorithms and models of the admission score of the colleges and universities based on machine learning methods do not take such competitive relationship into consideration, but simply make predictions for individual college or university, causing low predication accuracy and poor generalization capability. This paper intends to analyze such competitive relationship by extracting the important features (e.g., project, location and score discrepancy) of colleges and universities. A novel competition model incorporating the coarse clustering is thus proposed to make the predictions for colleges and universities in a same cluster. By using Gaokao data of Shanxi province in China from 2016 to 2019, we testify the proposed model in comparison with several benchmark methods. The experimental results show that the precision within the error of 3 points and 5 points are 7.3% and 2.8% higher respectively than the second-best algorithm. It has proven that the competition model has the capability to fit the competitive relationship, thus improving the predication accuracy to a large extent. Theoretically, the method proposed could provide a more advanced and comprehensive view about the analysis of factors that may influence the admission score of higher institutions. Practically, the model proposed with high accuracy could help the students make the best of their scores and apply for the college and universities more scientifically.

## Introduction

As one of the most significant talent selection systems in China, Gaokao is the primary way for high school graduates (i.e., candidates) to enter colleges and universities [1, 2]. The conditions that determine whether candidates can be admitted to their ideal colleges and universities are not only their scores, but also their decision of filling in parallel applications. After knowing the scores of the college entrance examination, a candidate could start to fill in the parallel application by choosing multiple proper colleges and universities [3–8]. Then, according to the number of (annual) enrollment plan, each college and university will select the candidates based on their scores. The lowest score of candidates for admission of one college or university is called its admission score. If a student’s first application fails, he will be transferred to the second application. In other words, students usually put good colleges at the top of their applications and the other options at the bottom. In this way, they not only have the opportunity to get admitted to a better university, but also have a guaranteed choice. Every year, the admission scores of colleges and universities are affected by a series of factors, such as the changes of the number of enrollment plan, the distribution of Gaokao scores, and the national admission policy, and thus fluctuate within a certain range. For a candidate, the accurate prediction of admission scores of colleges and universities is a big challenge, adding much difficulties to the reasonable use of the Gaokao score for applying for ideal colleges and universities.

Most candidates use some traditional algorithms to roughly predict the admission scores of colleges and universities. The equivalent score (ES) algorithm and the score difference (SD) algorithm are two widely-used methods for the benchmarks, which can roughly predict the admission score of individual college and university. With the development of date mining technology, some simple machine learning models were adopted for the prediction of the admission scores of colleges and universities. For example, the regression analysis model is used to predict the current admission score via historical ones [9], and the neural network model is adopted to construct the prediction model of admission scores [10].

**Fig 1:**
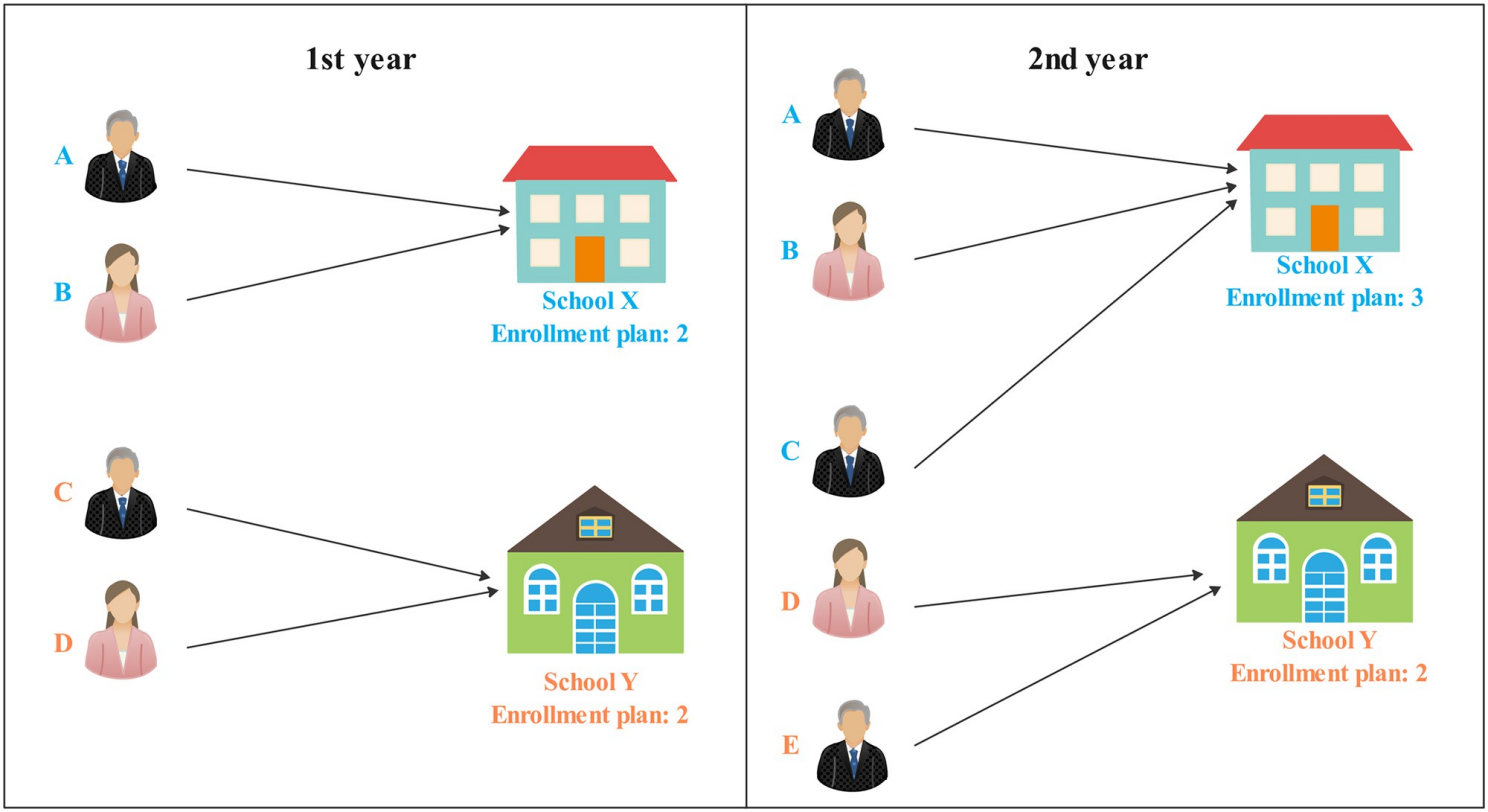
The schematic description of the competitive relationship between college X and Y. The changing number of enrollment plan of college X from the 1st year to the 2nd year results in the decreasing admission score of college Y in the 2nd year.

The existence of the competitive relationship among colleges and universities entails much attention when predicting the admission scores of these higher institutions. It is mainly caused by the rules of filling in parallel application. The number of the enrollment plan or the changes in the enrollment majors of one college also affects the admission scores of other colleges. Suppose that there are two colleges with similar comprehensive strengths (See Fig 1), namely, college X and college Y, but X is slightly better than Y. For the first year, four candidates A, B, C, and D whose scores are in a descending order with small gaps, have filled in X and Y at the same time. Because the priority of X is higher than that of Y, students A and B with higher scores are admitted to the X, so that students C and D can only be admitted to Y. For the second year, college X expanded the number of enrollment plan to three persons, while that of Y remains unchanged. At this time, students A, B, and C can be admitted to college X, and student D to college Y. And there is one enrollment place left at college Y, so that students E with a lower score than student D can be admitted to college Y. Thus, the change of enrollment plan of college X indirectly affects the admission score of college Y. However, there are still many unsolved problems regarding the prediction of admission scores, which can be summarized as follows:

- Some prediction models (or algorithms) rely on long-term historical data, but the annual changes of the college entrance examination and enrollment policy will devalue data collected many years ago.
- In the modeling process, the predication models directly uses the previous year’s admission score (or its corresponding enrollment rank according to the provincial score-rank comparison table as the feature of the college and university, and the current year’s admission score or its corresponding provincial rank as its label. However, there are not many colleges and universities having the same score section. It leads to the sparseness of training data and the decrease of the prediction accuracy.
- The competitive relationship among colleges and universities is general in Gaokao. The existing prediction models completely neglect this critical factor.

In order to solve the aforementioned problems, we have proposed a competition model for prediction of admission scores of colleges and universities. The competition model doesn’t need long-term historical data to built prediction model, but only uses four-year-phase (i.e., the four years before the prediction year) history data. The competitive relationship is comprehensively considered by extracting important features, such as the brand, location and score discrepancy. The building of competition model includes two consecutive steps, that is, the first step is the preliminary prediction of enrollment rank and the second step is the fine prediction of enrollment rank based on the competitive relationship. In the first step, the colleges and universities are divided into the stable and unstable set according to the stability of their four-year-phase admission scores, and their preliminary enrollment ranks in the prediction year are predicted by the ES algorithm and the gradient boosted decision tree (GBDT) model [11–13], respectively. In the second step, the proximate colleges and universities are clustered based on the features of the competitive relationship by the K-Means algorithm, and they are sorted in each cluster for the fine prediction of enrollment ranks. The more details of the competition model will be illustrated in Section “Prediction Model”.

First, the traditional algorithm of the equivalent score algorithm is used, which could determine the approximate range of the predicted value, and then combine the regression model to narrow the range of the predicted value. Additionally, we construct the competitive model upon this method, which is not considered by other previous methods. The competitive model can well fit the competitive relationship between multiple institutions, thereby further reducing the prediction error and improving the accuracy. At the same time, the introduction of the competitive model can improve the generalizability of the model. In short, we summarize the contributions of this paper:

- The competitive relationship among colleges and universities are firstly introduced to construct the prediction model of admission score in Chinese Gaokao. We use diverse features of colleges and universities to characterize their novel competition relationships and build competition model based on K-Means algorithm.
- We propose the novel two-step competition model for prediction of admission scores of colleges and universities. In the modeling process, the proposed model solves the non-trivial problems that have not been considered in the previous prediction model. For examples, we divide colleges and universities into stable set and unstable set according the fluctuations of short-term historical data, and use two kinds of prediction models for the preliminary prediction of enrollment rank. We use the change rate of enrollment rank as a label to train the GBDT model and preliminarily predict the enrollment rank of colleges and universities in unstable set. It avoids the sparseness of training data to a great extent, and improve the prediction accuracy of the GBDT model.
- The competition model is used to consecutively predict the preliminary and fine enrollment rank, which helps us to obtain the predicted admission scores of colleges and universities. Meanwhile, we also verify the algorithmic stability of the competition model by varying with the change of parameters. The experimental results show the parameter insensitivity of the competition model.

The remainder of this paper is organized as follows. The related works are briefly discussed in Section “Related Works”. The model statement is introduced in Section “Prediction Model”. The experimental design and results are presented in Section “Prediction Model”. The experimental design and results are presented in Section “Results”. Finally, the conclusion is presented in Section “Conclusion”.

## Related works

This section briefly illustrates the related works on the factors concerning centralized university admission system and the prediction model of admission score of colleges and universities in Chinese Gaokao.

The past few years have witnessed the analysis of the factors concerning centralized university admission system, which is prevailing in many countries. Under this condition, students with limited choices of universities should be sufficiently risk-averse (Hernandez-Chanto, (2020) [14]. Using an economic perspective, Chade et al (2014) [15] were focused on the behavior of students and have developed a decentralized Bayesian model of college admissions. Other researchers allowed for the heterogeneity in students’ preferences for college attributes during the admission process, where the spatial distribution of college was identified as an important factor (Fu et al., 2022 [16]). Chen et al. (2018) [17] have found whether a college is prestigious and students’ concerns about their admissions outcomes as two important factors. Other important features include subsidies, disclosure of students’ priorities, and affirmative policies (Artemov, 2021 [18]).

Within the specific context of China, many scholars made impressive effort in investigating the admission lines of the college entrance examination. Wang and Shi (2017) [19] used a type of ensemble learning, namely, Adaboost algorithm to study and forecast the admission lines of the college entrance examinations in China, achieving acceptable performance. Guo [20] proposed an improved back-propagation neural network prediction model that conforms to the variation of pass marks on the college entrance examinations. Lu [21] calculates the admission probability of each college based on the normal distribution of candidates’ scores, and recommends the most suitable college for students based on the probability. Using the weakening factor and the new background value, Chen [22] improved the GM (1,1) model to predict the admission line of schools and majors, established a normal sample model, and predict the probability of admission. Ren [23] analyzed a series of factors that affect the admission score (i.e., year, province, major, and batch line)and built a neural network model. Zhang [24] combined the six simulated test scores and used SVM to build a model to predict candidates’ scores in the college entrance examination. Wu [25] used neural network to predict the admission scores of Chinese-foreign cooperative education majors, and solved the hotly-detated issues of “low frequency and high risk”. In this analysis, the college was regarded as the feature of major sample, and then normalized all features to eliminate the influence of dimensions between features. Zhang [26] built a mathematical prediction model instead of a machine learning model by using the admission score and score-rank table over the years, and improved the General Morphological Analysis to recommend colleges to candidates. According to the batch line and the highest score, Bian [27] converted the score into a percentage, thereby eliminating the influence of changes in the number of candidates with same score and the corresponding ranking of score. In addition, the regression model was used to predict admission score. Xu [28] constructed a model with a decision tree, and calculated the most important features using the least square method, and used four evaluation methods to evaluate the accuracy of the model. Wang [29] used Adaboost to design the prediction model. The selected features included the total number of candidates, the number of enrollment plans of all colleges, and the difficulty of the examination questions. And Random forest was used to obtain the contribution of each feature in the model. However, lack of the consideration of the competitive relationship between these higher institutions, the performance of these models could be negatively affected.

## Prediction model

**Fig 2:**
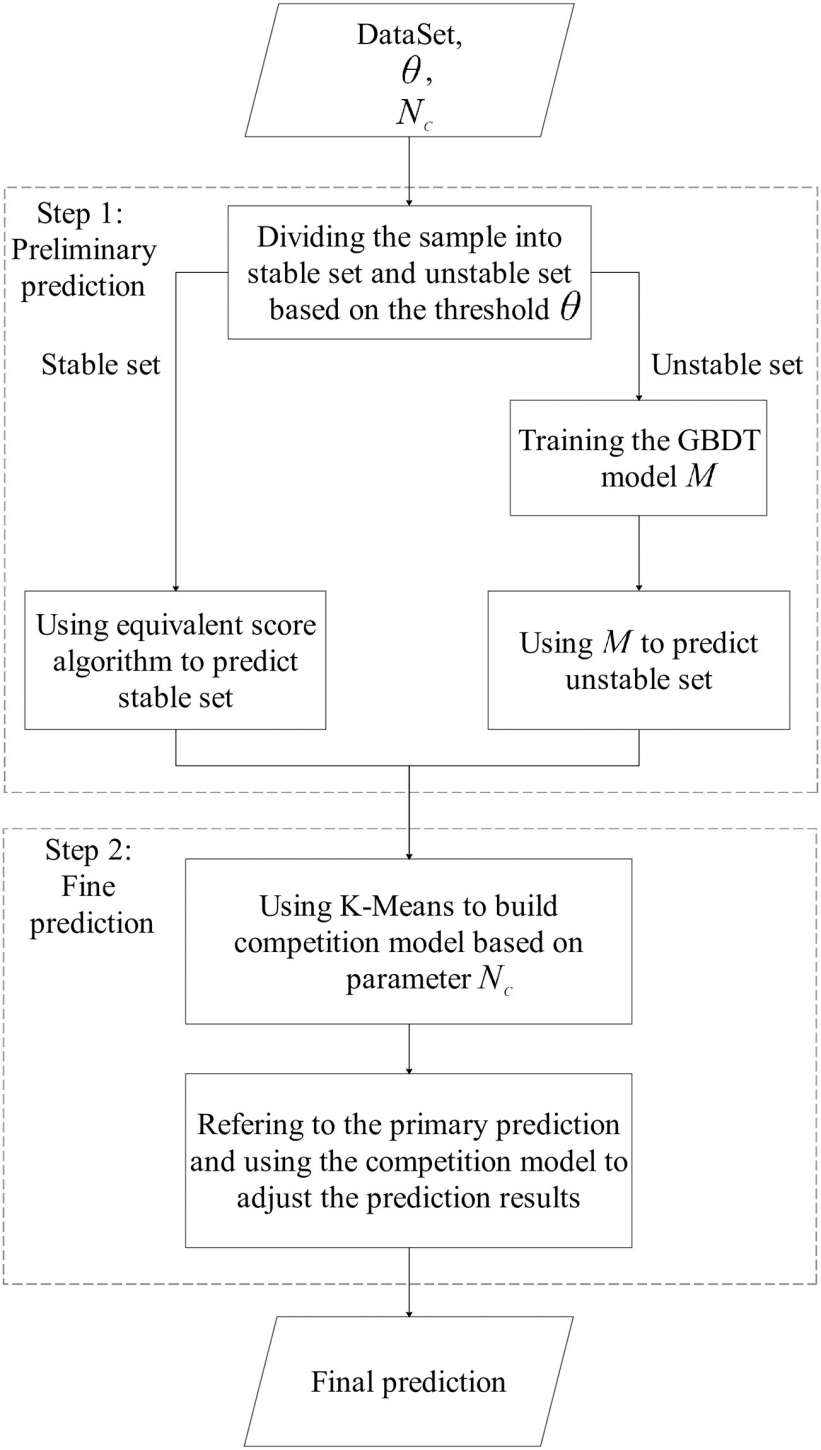
The flowchart of the competition model. It divides the framework into two consecutive steps. In the first step, the task is the preliminary prediction of enrollment rank, and in the second step, the task is the fine prediction of enrollment rank. The predicted admission score is translated from the predicted enrollment rank according to the provincial score-rank comparison table.

In this section, we mainly introduce the competition model and illustrate its framework in detail. Fig 2 shows the flowchart of the competition model, which clearly divide the framework into two consecutive steps. In each step, the task is realized by the crucial algorithms. We will present the detailed descriptions of these algorithms to keep the competition model as self-contained as possible. Additionally, the problem definition of the prediction of admission score is quite simple and clear, so that we only summarize the descriptions of symbols (or parameters) in the competition model, which is shown in Table 1.

**Table 1:** The notation of each symbol.

Symbol 1	Description
*s*_*t*_	the admission score in year *t*
*r*_*t*_	the enrollment rank in year *t*
*d*_*t*_	the score difference in year *t*
*b*_*t*_	the batch line in year *t*
*m*_*s*2*r*_(*t*, *s*)	the score to enrollment rank mapping function
*m*_*r*2*s*_(*t*, *r*)	the enrollment rank to score mapping function
*θ*	the threshold
${\hat{s}}_{t}$	the predicted admission score
*S*	the stable set
$\bar{S}$	the unstable set
*δ*_*k*_	the difference of equivalent score
*φ*_*k*_	the equivalent score
*j*_*n*_	the number of enrollment plans
*z*_*n*_	the number of majors
*γ*_*p*_	the rate of change in the number of enrollment plans
*γ*_*m*_	the rate of change in the number of majors
*γ*_*rank*_	the rate of change in the enrollment rank
$s_{max}^{\left(n-1\right)}$	the highest major admission score
$s_{min}^{\left(n-1\right)}$	the lowest major admission score
Δ	the score discrepancy
*N*_*c*_	the number of clusters
*P*	the set of the preliminarily predicted enrollment rank
*Q*	the set of the enrollment rank before the prediction year
$\hat{S}$	the final prediction of admission score for every university

### Preliminary prediction

Under the combined effect of various factors, we find that the admission scores of some colleges and universities annually fluctuate within a small range. That is, a portion of colleges and universities are relatively stable in predicting their admission scores. We classify them into the stable set *S* if their fluctuation differences between their own equivalent scores are not more than three points, and use the ES algorithm to realize the preliminary prediction. The remainders are classified into the unstable set $\bar{S}$, and the preliminary prediction is realized based on the GBDT model according to the change rate of the enrollment rank.

Suppose that there are the Gaokao data of *n* consecutive years. We firstly transform the admission scores into the equivalent scores according to the provincial score-rank comparison table of the current year and (*n* + 1)-th year (i.e., the prediction year). For each sample (i.e., college or university), the fluctuation differences *δ*_*k*_ of the equivalent scores are then computed, defined as *D* = {*δ*_2_, *δ*_3_, …, *δ*_*n*_}. If ∀*δ* ∈ *D* satisfies |*δ*| ≤ 3, otherwise it will be in set $\bar{S}$. The sample division algorithm is shown in Algorithm 1.

**Algorithm 1** Sample division

**Input** admission score *s*_*i*_ (*i* = 1, 2, …, *n*) from 1-st year to *n*-th year, and threshold *θ*.

**Output** *label* = 1 if it’s a stable sample else *label* = 0.

1: **for** *i* = 1, 2, …, *n* **do**

2:  Getting admission score *s*_*i*_ of i-th year;

3:  Getting corresponding enrollment rank *r*_*i*_ of *s*_*i*_ through the provincial score-rank table of *i*-th year, i.e., *r*_*i*_ = *m*_*s*2*r*_(*i*, *s*_*i*_);

4:  Calculating equivalent score *φ*_*k*_ through the provincial score-rank table of (*n* + 1)-th year, i.e., *φ*_*k*_ = *m*_*r*2*s*_(*n* + 1, *r*_*i*_).

5: **end for**

6: **for** *k* ∈ [2, *n*] **do**

7:  Calculating *δ*_*k*_ = *φ*_*k*_ − *φ*_*k*−1_;

8:  **if** *δ*_*k*_ > *θ* 0 **then**

9:   **return** 0

10:  **end if**

11: **end for**

12: **return** 1;

After the sample division, the preliminary prediction in the stable and unstable sets, respectively. For the samples in the stable set, we directly use the ES algorithm to preliminarily predict the admission score (or the enrollment rank) because of its higher accuracy. While for the samples in the unstable set, we need to consider the combined effect of various factors, and select more significant features into the regression analysis of preliminary prediction. For example, the sensitivity to the changes of the number of enrollment plan and in enrollment majors is diverse. Furthermore, the admission score discrepancy, namely the difference between the highest and lowest admission scores of a sample is an important feature to implicitly measure the degree of sensitivity. The smaller the admission score discrepancy is, the more similar the level of candidates who select the sample are, and the less likely the admission score to be affected. After we determine these important features, what we consider is the use of regression model to fit the fluctuation of college admission scores. Herein, the GDBT model is used for regression analysis.

Let the training data set be the historical data of samples in the (*n* − 1)-th year and the *n*-th year, and the test data set be the historical data of samples in the *n*-th year and the (*n* + 1)-th year. In the process of training preliminary prediction model, the following features of each sample need to be extracted,

$$\gamma_{p}=\frac{j_{n}}{j_{n-1}},$$ (1)

$$\gamma_{m}=\frac{z_{n}}{z_{n-1}},$$ (2)

$$\Delta ={s_{max}}^{\left(n-1\right)}-{s_{min}}^{\left(n-1\right)}.$$ (3)

*j*_*n*_ represents the number of enrollment plan of the sample in the *n*-th year, and *γ*_*p*_ represents the relatively changing rate of the number of enrollment plan of the sample; *z*_*n*_ represents the number of enrollment majors of the sample in the *n*-th year, and *γ*_*m*_ represents the relatively changing rate of the enrollment majors of the sample; ${s_{max}}^{\left(n-1\right)}$ and ${s_{min}}^{\left(n-1\right)}$ represent the highest and lowest major admission scores of the sample in the (*n* − 1)-th year, and Δ represents the score discrepancy of the sample.

The label value of the sample needs to be expressed by the rate of change in admission scores. Considering that the difficulty of the college entrance examination changes each year, the scores of the same rank are not the same, so that the score cannot be representative of the true level of admission score. To this regard, the results should be measured by the corresponding rank in the provincial score-rank table. In the process of training regression model, we set the label value of the sample

$$\gamma_{rank}=\frac{r_{n}}{r_{n-1}}.$$ (4)

*r*_*n*_ represents the enrollment rank of the sample in the *n*-th year, and *γ*_*rank*_ represents the change rate of the enrollment rank of the sample.

Through the features and label value calculation method above, the input of training the GBDT model could be regarded as (*x*_*i*_, *y*_*i*_), where *x*_*i*_ = (*γ*_*p*_, *γ*_*m*_, Δ), *y*_*i*_ = *γ*_*rank*_. We extract the features of the (*n* − 1)-th year and the label values of the *n*-th year respectively, and input them into the GBDT model to obtain the trained model *M*. After that, we extract the features of the *n*-th year and use the trained model *M* to obtain the preliminary prediction of ${\hat{\gamma }}_{rank}$. Then, according to the Eq (4), we use the enrollment rank of the *n*-th year to obtain the enrollment rank of the (*n* + 1)-th year, ${\hat{r}}_{n+1}={\hat{\gamma }}_{rank}\times r_{n}$.

Taking the changing features of the colleges and universities themselves into consideration, the ES algorithm and the trained model *M* are used to predict ${\hat{r}}_{n+1}$ of each sample in *S* and $\bar{S}$ respectively. The set *P* of the preliminarily predicted enrollment rank ${\hat{r}}_{n+1}$ of all samples is obtained. Note that the admission score ${\hat{s}}_{n+1}$ of each sample can be transformed from the ${\hat{r}}_{n+1}$ according to the provincial score-rank table of the (*n* + 1)-th year.

### Fine prediction

The second step is to blend the competitive relationships among colleges and universities into the fine prediction of enrollment rank. The main content of this section is to analyze the critical factors influencing the competition among colleges and universities and improve the competition model.

An important feature to analyze whether there are competition among colleges and universities is the overlapping degree of admission scores, that is, the overlapping degree of their highest and lowest scores. The highest and lowest scores of college (or university) *A* (*B*) are $s_{max}^{A}$ ($s_{max}^{B}$) and $s_{min}^{A}$ ($s_{min}^{B}$), respectively. Supposing $s_{max}^{A}>s_{max}^{B}>s_{min}^{A}>s_{min}^{B}$, $s_{max}^{B}-s_{min}^{A}$ is the overlapping degree. If the overlapping degree is larger, it means that the more similar the level of candidates admitted by the two colleges is, the greater the degree of competition between these two colleges. On the contrary, if the overlapping degree is less than 0, it means that there is almost no competition between them. For example, through the statistical analysis of historical data of Tsinghua University and Peking University, we can find that they have a high overlapping degree, whereas their overlapping degree between the two and other excellent universities (e.g., Shanghai Jiaotong University, Fudan University, Renmin University of China, and Zhejiang University) is less than 0. It means that the competition between Tsinghua University and Peking University is large, but the competition between the first two and the latter four is negligible.

Meanwhile, the competition can be transmitted. For example, the overlapping degree between College *A* and College *B* is relatively large, and the overlapping degree between College *A* and College *C* is less than 0, but there is the relatively large overlapping degree between College *B* and College *C*. Such case suggests that college *A* indirectly competes with college *C*. Relatively speaking, this kind of indirect competition is caused by the competition between College *A* and College *B*. Though theoretically weaker than direct competition, it should still be taken into consideration.

Besides the overlapping degree, there are a series of features that affect the competition between colleges and universities, including the brand and geographic location. On the one hand, the brand includes universities of the Project 985, universities of the Project 211, and the newly-classified ‘double-first-class’ universities in recent years. On the other hand, the geographic location is also an important feature that the candidates need to consider. Colleges and universities in first-tier cities such as Beijing, Shanghai and Guangzhou, are favored by candidates. Therefore, they are more likely to compete each other. Similarly, such case can also occur in non-first-tier cities, thus the geographical location of colleges and universities is also an indispensable important feature. In short, the features used in the competition model are summarized as: 1) the highest score, 2) the lowest score, 3) whether it’s in a firs-tier city, 4) whether it belongs to Project 985, 5) whether it belongs to Project 211, 6) whether it belongs to ‘double-first-class’ program.

In further, the direct and indirect competition are obviously enhanced in a subset of colleges and universities that are clustered based on their features. The core idea of the competition model is to divide colleges and universities with the stronger competition into the same subset. Alternatively, the competition model for the fine prediction use the K-Means clustering algorithm [30, 31] to divide the entire set of colleges and universities into multiple subsets. In each subset, we sort the colleges and universities according to their enrollment rank. Then, two new sequence sets $P_{c}=\{p^{\left(1\right)},p^{\left(2\right)},\ldots ,p^{\left(N_{c}\right)}\}$ and $Q_{c}=\{q^{\left(1\right)},q^{\left(2\right)},\ldots ,q^{\left(N_{c}\right)}\}$ are obtained. It is obvious that *p*^(*i*)^ and *q*^(*i*)^ correspond to cluster *c*_*i*_. As *p*^(*i*)^ and *q*^(*i*)^ are ordered, for any *u* in cluster *c*_*i*_, the index of *u* in *p*_*i*_ is *j*, but the *j*-th college (or university) of *q*_*i*_ may not be *u*. This is because the competitiveness of *u* is different in *n*-th and (*n* + 1)-th year. The *j*-th college in *q*_*i*_ has close competitiveness with *u*, so the average of the two enrollment rank is more accurate. The final predicted admission score is transferred from the finely predicted enrollment rank according to the provincial score-rank comparison table. The more details on the computational procedure is shown in Algorithm 2.

**Algorithm 2** Competition Model

**Input** number of clusters *N*_*c*_, preliminarily predicted enrollment rank set of (*n* + 1)-th year *P*, enrollment rank set of the of (*n*)-th year *Q*.

**Output** Final prediction of admission score set for every university $\hat{S}$

1: Using K-Means to cluster all universities with parameter *N*_*c*_, and get a partition $C=c_{1},\;c_{2},\;\ldots ,c_{N_{c}}$;

2: Sorting universities in every cluster *c*_*i*_(*i* = 1, 2, …, *N*_*c*_) according to *Q* and *P* respectively, and get two sequence sets $Q_{c}=\{q^{\left(1\right)},q^{\left(2\right)},\ldots ,q^{\left(N_{c}\right)}\}$ and $p_{c}=\{p^{\left(1\right)},p^{\left(2\right)},\ldots ,p^{\left(N_{c}\right)}\}$;

3: **for** *u* ∈ *c*_*i*_, *i* ∈ [1, *N*_*c*_] **do**

4:  Getting index *j* of *u* in *p*^(*i*)^;

5:  Final predicted enrollment rank ${\hat{r}}_{u}=\frac{q_{j}^{\left(i\right)}+p_{j}^{\left(i\right)}}{2}$;

6:  Getting final predicted admission score using the enrollment rank to score mapping function *m*_*r*2*s*_(*t*, *r*), i.e. ${\hat{s}}_{u}=m_{r2s}\left(n,{\hat{r}}_{u}\right)$.

7:  Add ${\hat{s}}_{u}$ to $\hat{S}$;

8: **end for**

9: **return** $\hat{S}$.

## Result

The contrast experiments are designed based on benchmark prediction algorithms (i.e., ES, SD, and linear regression (LR) model) and two up-to-data algorithms (i.e., weight slicing model (WSM) and weighted point model (WPM) [26]). They validate the effectiveness of the competition model for the admission score prediction of colleges and universities. In this section, we briefly illustrate the basic information of dataset, the principles of the benchmark methods, the evaluation index, the analysis of parameters, and analysis of accuracy, respectively.

### Dataset

This research mainly uses the historical Gaokao data of 1A colleges and universities in Shanxi province from 2016 to 2019. The data we used was collected from the official website of Shanxi Province, which boasts high reliability and good quality. The sample of the data used in our analysis could be downloaded from the official website of Shanxi Examination Institute (URL: http://www.sxkszx.cn/news/ptgk/index_3.html). The complete dataset is available from the authors upon reasonable request. The data is indexed by the name of the college and the name of the major, including the lowest admission score of the major, the highest admission score of the major, the enrollment plans of the major, the collage brand (e.g., the 985 project, the 211 project, and the double-first class levels) and the location of the collage. There are about 3,000 samples each year, including an average of 151 colleges and more than 18,000 enrollment plans. The items contain school name, major name, enrollment plans of the major, and the highest and lowest scores of the majors. The admission score of a college (or university) equals to the lowest score across all majors. This short-term Gaokao data is divided into two parts: the one of 2016 to 2018 is used for training model, and the one of 2019 is used for testing model. We herein don’t consider the prediction of the admission score of the majors because the majors are apparently different among colleges and universities.

### Benchmark methods

Herein, we mainly illustrate the principles of ES and SD. The principles of other methods are presented in their previous work, which is directly referred to the reference [26]. In the following, we briefly introduce ES and SD.

**ES**: Each score can correspond to the rank through the provincial score-rank table. Due to the different difficulty of the examination each year, the admission score of the same school may be very different for two years, but the corresponding rank changes corresponding to the score line are very small, so the equivalent score algorithm was proposed. Let *r*_*t*_ = *m*_*s*2*r*_(*t*, *s*) be the mapping from score to rank at year *t*, where the *s* represents score. Let *s*_*t*_ = *m*_*r*2*s*_(*t*, *r*) be the mapping from rank to score at year *t*, where the *r* represents rank. Then, equivalent score of year *t*_1_ relative to year *t*_2_ can be expressed as

$${\hat{s}}_{t_{1}}=m_{r2s}\left(t_{2},m_{s2r}\left(t_{1},s_{t_{1}}\right)\right).$$ (5)

Due to the small fluctuations in some colleges and universities, the accuracy is higher than most algorithms, but various influencing factors are ignored, and all colleges and universities are treated as a static whole, resulting in large prediction errors.

**SD**: The admission of college entrance examination is generally divided into multiple batches. The higher the admission batch, the higher the grade. Each batch has a corresponding batch line, and only candidates whose scores are higher than the batch line are eligible to fill in the corresponding parallel application. The discrepancy between the admission score and the batch line is called the score difference. Given the admission score $s_{t_{1}}$ of one college (or university) and the batch line $b_{t_{1}}$ in year *t*_1_, the score difference is $d_{t_{1}}=s_{t_{1}}-b_{t_{1}}.$ Then, the prediction score in year *t*_2_ with batch line $b_{t_{2}}$ can be calculated:

$${\hat{s}}_{t_{2}}=d_{t_{1}}+b_{t_{2}}$$ (6)

### Evaluation

Since the prediction model is only a prediction for the admission scores of colleges and universities, traditional evaluation indicators such as MSE (mean square error), MAE (mean absolute error), and RMSE (square root error) cannot intuitively predict the accuracy of the model, but evaluate the rate. Therefore, we need a new evaluation index.

According to the college admission score prediction experience, it is stipulated that if the absolute value of the discrepancy between the sample’s actual admission score and the predicted one $\epsilon =|\hat{s}-s|\leq 3$, the predicted admission score can be considered as accurate; if 3 < *ϵ* ≤ 5, the predicted admission score can be considered to be relatively accurate; if 5 < *ϵ* ≤ 7, the predicted admission score can be considered not accurate enough, but still has a strong reference value; if *ϵ* > 7, the predicted admission score is considered to have a large error and has no reference value. Then, the precision of model can be calculated as

$$p_{i,j}=\frac{Count(i<\epsilon \leq j)}{N},$$ (7)

where *N* refers to the sample size. Using the above measurement criteria to evaluate the model prediction results, we need to not only pay attention to the proportion of colleges with an error of *ϵ* ≤ 7, but also focus on the proportion of colleges with an error of *ϵ* ≤ 3.

### Parameter analysis

**Fig 3:**
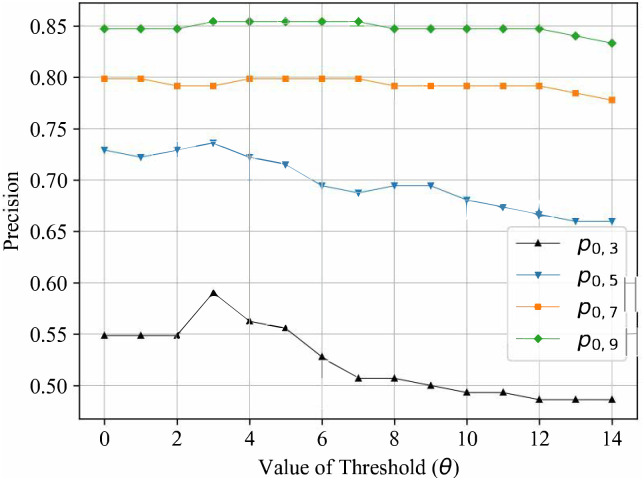
The precision within each error range versus *θ*.

**Fig 4:**
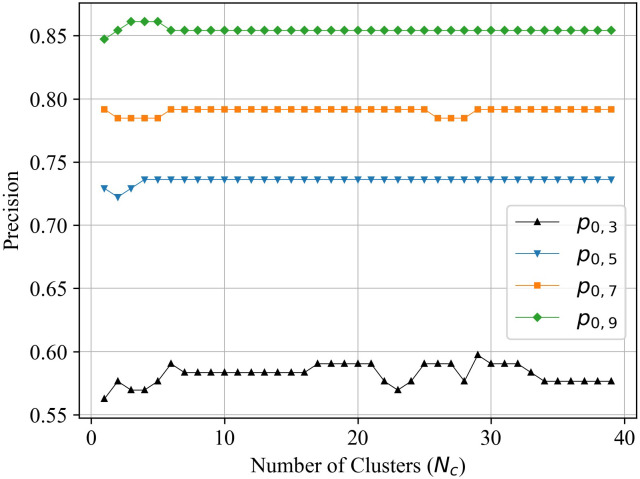
The precision within each error range versus *N*_*c*_.

According to the statements of the competition model, there are two key parameters, *θ* in the preliminary prediction and *N*_*c*_ in the fine prediction. Both of them are non-zero positive integers, so the parameter tuning process is relatively simple. By fixing one parameter and adjusting another parameter, we can obtain the precision varies with *θ* and *N*_*c*_, respectively. In Fig 3, we present the precision *p*_0,3_, *p*_0,5_, *p*_0,7_, *p*_0,9_ versus *θ* when setting *N*_*c*_ = 29. It can be seen that the optimal values is *θ* = 3 according to *p*_0,3_. In Fig 4, we present the precision *p*_0,3_, *p*_0,5_, *p*_0,7_, *p*_0,9_ versus *N*_*c*_ when setting *θ* = 3. It can be seen that the optimal values is *N*_*c*_ = 29 according to *p*_0,3_. Meanwhile, we note that the precision is more sensitive to *θ*. Too large *θ* makes more colleges and universities be divided into stable set, and they are preliminarily predicted by the ES algorithm. Thus, the effectiveness of the competition model is weakened to some extent. When tuning the parameter *N*_*c*_, *p*(0, 3) continues to change and reaches the optimal number of the cluster. It suggests that clustering colleges and universities in the fine prediction is of great importance in precisely acquiring the competition among them.

### Accuracy analysis

**Fig 5:**
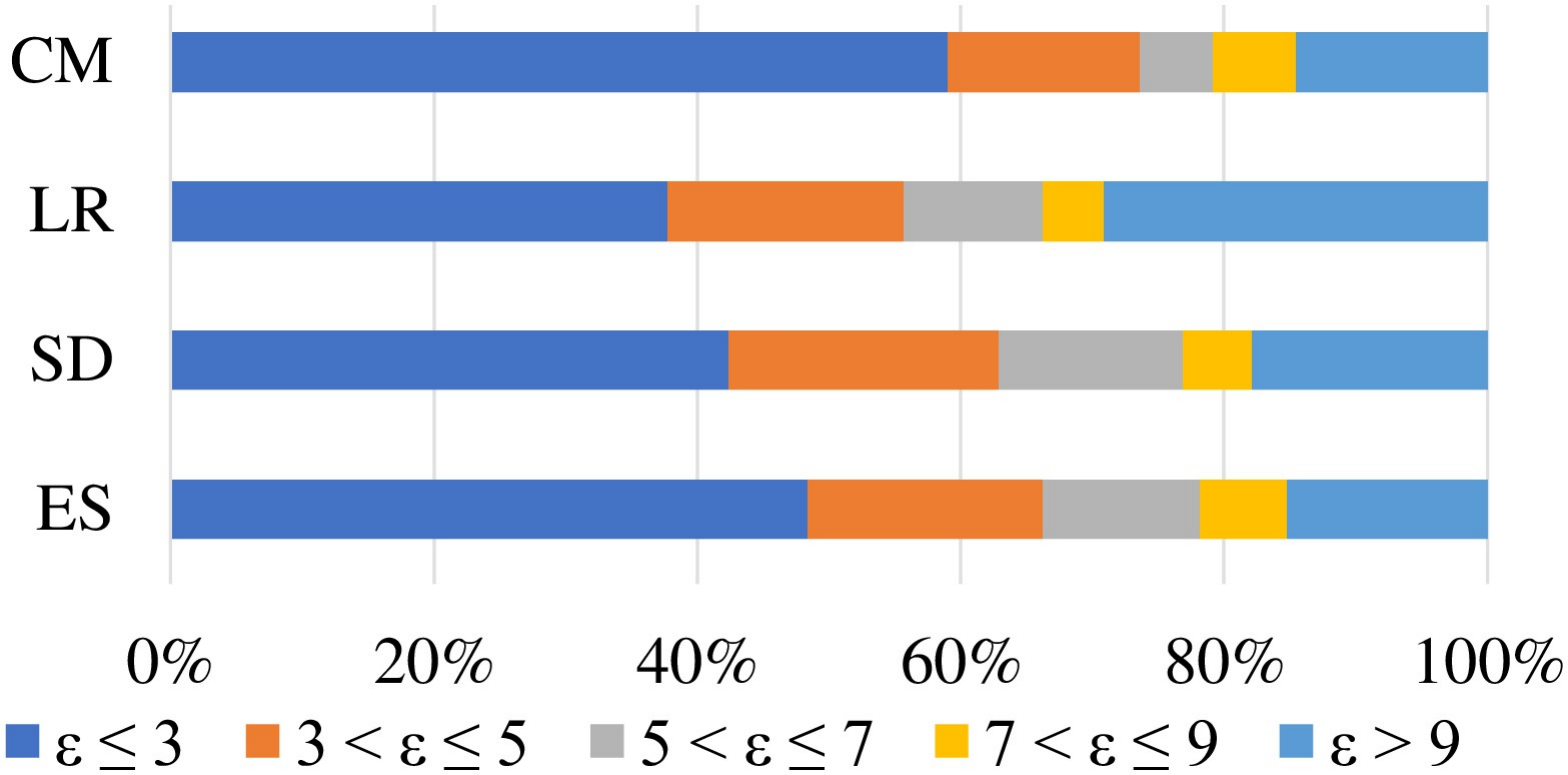
Comparison of the competition model to five contrast methods.

After fixing the optimal values of *θ* and *N*_*c*_, we present the comparison of the competition model to five contrast methods in Fig 5. It can be seen that *p*_0,3_ (i.e., *ϵ* ≤ 3) obtained from the competition model is the highest, of which the value is 0.597. It exceeds the second-best value of the WSM more than 7.4%. The high precision of the competition model suggests that it can predict the admission scores of nearly 60% colleges and universities within *p*_0,3_. In further, we present accumulate precision by enlarging error range, and the experimental results are shown in Table 2. These experimental results show that the competition model can correct the admission scores with little volatility and improve the overall accuracy through the analysis of competition relationships. For colleges and universities with large fluctuations, their unquantifiable factors cannot be all introduced into the competition model, resulting in a close proportion of the competition model, the WSM and the WPM when enlarging the error range (e.g., *ϵ* ≤ 7).

**Table 2:** The accumulative accuracy rate of the competition model and five contrast methods.

	*p*_0,3_	*p*_0,5_	*p*_0,7_	*p*_0,9_
ES	0.483	0.662	0.781	0.848
SD	0.424	0.629	0.768	0.821
LR	0.378	0.556	0.662	0.709
WSM	0.556	0.716	0.796	0.840
WPM	0.525	0.698	0.802	0.839
CM	0.597	0.736	0.792	0.854

Taking the competition relationship into consideration, the competition model can be regarded as a more sophisticated model. It can better reflect the real situation, thus producing a more satisfactory prediction results. In addition, since the competition relationship exists in most of the colleges and universities in the world other than in China, the incorporation of the competition relationship could provide insights for future analysis on the prediction of the admission scores and conditions in other higher institutions in the world.

## Conclusion

In conclusion, it is of importance to correctly understand and summarize the law of the prediction of college entrance examination results, and it is the key to improve the accuracy of prediction. The theoretical importance of this research is as follows. First, our research provides an innovative perspective that all colleges and universities interact with each other dynamically, and a competition model must thus be introduced to adjust the predicted value of all colleges and universities. Secondly, we concluded that direct prediction of college admissions results often fails to achieve good results, because the rate of change in admission scores is the core of the prediction. Indirect prediction of admission scores through the rate of change in admission scores can achieve fair results. Finally, it is found that when comprehensive factors in the admission process cannot be resolved, we should try to modify the prediction results based on traditional prediction algorithms to make them closer to the true value. For that, we provide the novel two-step competition model for prediction of admission scores of colleges and universities. And the experimental results based on the designed evaluation index show that it outperforms other contrast methods and has an application value in the real scenario. In particular, in the proposed model, the characteristic of the regression model (i.e., GBDT model) is to make small corrections on the basis of previous years’ admission results to obtain preliminary prediction of admission score. The competition model, however, is a larger revision of the preliminary prediction to make it closer to the actual admission score. The combination of the two can greatly improve the prediction accuracy. The practical implications of this research are presented. For students, our model could help them fill in their applications scientifically. Due to the particularity of Chinese college entrance examination admission, candidates can make the best use of their test scores and apply to the ideal university under the condition that they can predict the admission line. For the policy makers in higher institutions, they could use these findings as an important guidance to improve their own competitiveness to attract more high performers in the college entrance examination.

It should be noted that this research is not without limitations. First, considering the sensitivity of college entrance examination data, the data obtained is not complete, lacking information of many dimensions, especially comprehensive factors, so that the feature selection only included a few aspects. Secondly, due to the lack of data dimension, the competition model proposed in this research, to a certain extent, could not fit the competition between colleges completely. Future research is suggested to be carried out on more representative samples to improve the accuracy of the model. Third, the model only predicts the admission scores of colleges and universities, but does not predict the admission scores of majors. However, the admission scores of majors are also important. This issue needs to be taken into account in the follow-up work, and a major prediction model should be proposed on the basis of the prediction model of admission scores of colleges and universities, so as to provide a more accurate model for candidates to fill in the application. Fourth, contextual factors, including social environment, national policies, economic conditions and employment prospects were not incorporated in this model. For example, the admission scores of most economic colleges in Shanxi Province fell sharply in 2019. The Covid-19 epidemic in 2020 [32, 33] would affect the admission scores of universities in Hubei Province and some medical colleges. The college entrance examination big data only records the admission status and major information of each school over the years, and does not include these factors. It is believed that comprehensive factors will have an impact on colleges and universities, but there is no data or indicator to quantify these impacts, so we cannot use models to predict the degree of impact. We can only deduce it from our experience, to adjust the predicted value of the model, which is also a problem common to all current prediction models.

## Supporting information

S1 Dataset (ZIP)
